# Acute Presentation and Persistent Glomerulonephritis Following Streptococcal Infection in a Patient With Heterozygous Complement Factor H–Related Protein 5 Deficiency

**DOI:** 10.1053/j.ajkd.2012.02.329

**Published:** 2012-07

**Authors:** Katherine A. Vernon, Elena Goicoechea de Jorge, Angela E. Hall, Veronique Fremeaux-Bacchi, Timothy J. Aitman, H. Terence Cook, Robert Hangartner, Ania Koziell, Matthew C. Pickering

**Affiliations:** 1 Centre for Complement and Inflammation Research, Imperial College, London, United Kingdom; 2 Department of Immunology, Imperial College Healthcare NHS Trust, London, United Kingdom; 3 Service d'Immunologie Biologique, Hôpital Européen Georges Pompidou and INSERM UMRS 872, Cordeliers Research Center, Paris, France; 4 Physiological Genomics and Medicine Group, MRC Clinical Sciences Centre, Imperial College, London, United Kingdom; 5 Cellular Pathology, Guy’s and St Thomas' NHS Foundation Trust, London, United Kingdom; 6 Department of Paediatric Nephrology, Evelina Children's Hospital, Guy’s and St Thomas' NHS Foundation Trust, London, United Kingdom; 7 Department of Experimental Immunobiology, Division of Transplantation Immunology and Mucosal Biology, Kings College, London, United Kingdom

**Keywords:** Complement, kidney, streptococcus, C3 glomerulopathy

## Abstract

Acute poststreptococcal glomerulonephritis is a common cause of acute nephritis in children. Transient hypocomplementemia and complete recovery are typical, with only a minority developing chronic disease. We describe a young girl who developed persistent kidney disease and hypocomplementemia after a streptococcal throat infection. Kidney biopsy 1 year after presentation showed isolated glomerular complement C3 deposition, membranoproliferative changes, and subendothelial, intramembranous and occasional subepithelial electron-dense deposits consistent with C3 glomerulopathy. Complement gene screening revealed a heterozygous single nucleotide insertion in exon 4 of the complement factor H–related protein 5 gene *(CFHR5),* resulting in a premature stop codon. This variant was not detected in 198 controls. Serum CFHR5 levels were reduced. The mother and sister of the index patient were heterozygous for the sequence variant, with no overt evidence of kidney disease. We speculate that this heterozygous CFHR5 sequence variant is a risk factor for the development of chronic kidney disease after streptococcal infection.

Acute poststreptococcal glomerulonephritis (APSGN) is a common cause of childhood acute nephritis. Serum complement C3, C5, and properdin levels typically are reduced acutely, whereas C4 levels are normal.^1,2^ This pattern of hypocomplementemia reflects selective activation of the complement alternative pathway. Streptococcal components have been shown to activate this pathway,^3-5^ and transient C3 nephritic factor (C3NeF) has been shown in some cases.^6^ C3NeF potentiates activation of the alternative pathway and typically is seen in dense deposit disease, a condition strongly associated with alternative pathway dysregulation. In APSGN, most children recover completely and complement levels return to normal within 12 weeks. For reasons that are unknown, a minority develop chronic kidney disease associated with continuing hypocomplementemia.^7,8^ We report a case of persistent kidney disease after streptococcal infection in association with a heterozygous mutation in the gene encoding complement factor H–related protein 5 (*CFHR5*).

## Case Report

A previously healthy 7-year-old Caucasian female presented with a 10-day history of lethargy, fever, sore throat, and cough associated with abdominal pain, vomiting, and dark colored urine. Mild periorbital edema was accompanied by abnormal urinalysis results (protein [3+], blood [3+]). Results of investigations included the following values: serum urea nitrogen, 50.1 mg/dL (17.9 mmol/L); creatinine, 0.98 mg/dL (87 μmol/L); hemoglobin, 10.9 g/dL (109 g/L); C-reactive protein, 77 mg/L; C3, 0.36 mg/mL (0.36 g/L; reference range, 0.75-1.65); C4, 0.29 mg/mL (0.29 g/L; reference range, 0.11-0.43); and urine protein-creatinine ratio, 5,221 mg/g. Both serum anti-streptolysin O (>200 U/mL) and anti-DNase antibody titers (360 U/mL) were elevated. Kidney ultrasound showed 8.3-cm kidneys with loss of corticomedullary differentiation.

The patient improved after treatment with phenoxymethylpenicillin, although proteinuria and microscopic hematuria persisted. Four months later, she developed macroscopic hematuria after an upper respiratory tract infection.

**Figure 1:**
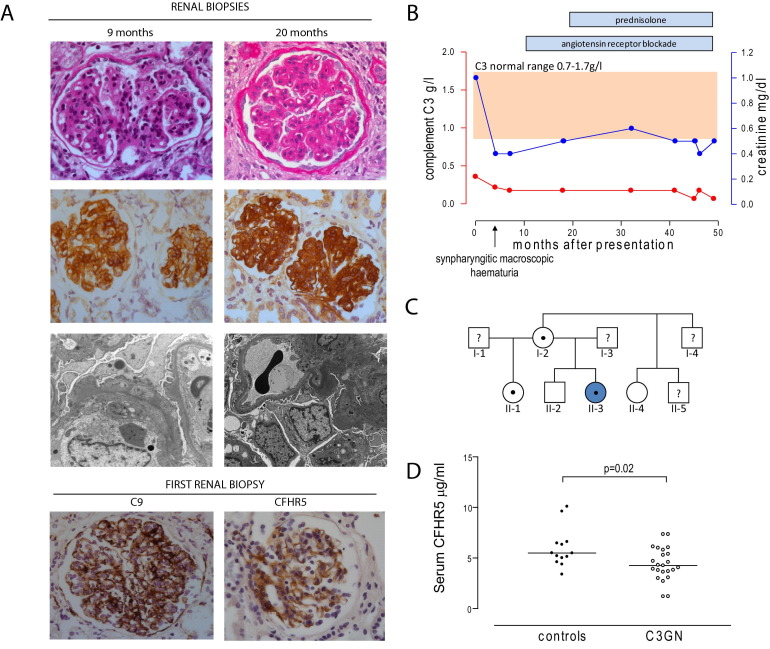
(A) Kidney biopsies performed at 9 and 20 months postpresentation. Light microscopy (first row) shows mesangial hypercellularity, segmental endocapillary hypercellularity, and capillary wall double contours with marked capillary wall C3, C9, and CFHR5 seen on immunoperoxidase staining (second [C3] and fourth [C9, CFHR5] rows). On electron microscopy (third row), there were intramembranous and occasional subendothelial electron-dense deposits. Rare hump-like subepithelial deposits were seen in the first biopsy specimen. Rabbit anti-human C3 (Dako, www.dako.com), mouse anti-human C9 (Leica, www.leica.com), and mouse anti-human monoclonal CFHR5 antibodies (a gift from Dr J. McRae) were used for immunoperoxidase staining. (B) Serum complement C3 and creatinine levels versus time. Serum C3 levels remained profoundly depressed throughout the illness. Conversion factor for serum creatinine in mg/dL to μmol/L, ×88.4. (C) Family pedigree. II-3 (filled circle) denotes the index patient. A central dot within the symbol (circle and square denoting female and male individuals, respectively) indicates the sequence variant is present, empty symbols denote absence of the sequence variant, and symbols with a question mark indicate genetic status unknown. (D) Serum CFHR5 levels in individuals with biopsy-proven C3 glomerulonephritis and absence of the CFHR5 sequence variant. Median CFHR5 level in the C3 glomerulonephritis group (4.3; range 1.2-7.4 μg/mL; n = 23) was significantly lower (*P* = 0.02, Mann-Whitney test) than the median in healthy controls (5.5; range 3.4-10.1 μg/mL; n = 13). CFHR5 was measured by enzyme-linked immunosorbent assay using rabbit anti-human CFHR5 and mouse anti-human CFHR5 antibodies (both from Abcam, www.abcam.com) as capture and primary antibodies, respectively. The standard curve was generated using recombinant CFHR5 (R&D Systems, www.rndsystems.com).

Nine months after presentation, kidney biopsy showed mesangial hypercellularity, segmental endocapillary hypercellularity, and segmental capillary wall double contours on light microscopy. Immunoperoxidase staining showed marked granular capillary wall and mesangial C3, C9, and CFHR5 deposition (Fig 1A). Glomerular C1q and immunoglobulins were absent. On electron microscopy, glomerular basement membranes appeared thickened, with segmental duplication of the basement membrane and mesangial cell interposition. There were intramembranous electron-dense deposits with occasional subendothelial deposits, as well as scattered hump-like subepithelial deposits. Mesangial deposits were absent. Angiotensin receptor blockade reduced urinary protein loss and kidney function remained normal.

Twenty months after presentation, a second kidney biopsy (Fig 1A) showed persistent membranoproliferative glomerulonephritis with tubulointerstitial scarring involving ∼40% of the cortex. Electron microscopy showed intramembranous electron-dense deposits and some mesangial deposits. The findings in both biopsies are consistent with C3 glomerulopathy with a membranoproliferative pattern of glomerulonephritis.^9^ Proteinuria improved with glucocorticoid therapy. Since the onset of disease, circulating C3 levels have remained low (Fig 1B). She has not developed ocular drusen or lipodystrophy. C3NeF has been consistently undetectable and anti-factor H autoantibodies have not been detected.

To determine whether there was any other serum factor enhancing C3 activation, we added purified C3 (0.5% solution; Merck, www.merck.com/index.html) to serum from the index case and compared its hemolytic activity at 2 and 4 hours with that of C3-deficient human serum reconstituted with purified C3 in an identical fashion. Hemolytic activity at 2 (60% vs 57%) and 4 hours (43% vs 47%) did not differ between the test and control sera, indicating that there was no evidence of accelerated serum C3 conversion in serum of the index case.

We performed screening for the known genetic causes of alternative pathway dysregulation. No coding mutations were detected in the complement genes CD46, complement factor H (CFH), factor B, factor I, and C3. No copy number variation within the *CFH-CFHR* gene locus was seen using a multiplex ligation-dependent probe amplification assay. CFHR5 gene sequencing revealed a single heterozygous nucleotide duplication in exon 4 (c.485dupA) which generates a reading frameshift at amino acid 163 and a premature stop codon at amino acid position 197 (p.Glu163Argfs*34). This variant was not detected by sequencing of 198 ethnically matched DNA samples (obtained from the UK Blood Services Collection of Common Controls) and was not present in dbSNP (www.ncbi.nlm.nih.gov/snp, accessed October 2011). The healthy mother (I-2) and sister (II-1), but not the 2 other siblings examined (II-2 and II-4), were heterozygous for this sequence variant (Fig 1C). The complement profile of the kindred is shown in Table 1. Serum CFHR5 levels in unaffected members with the gene variant were within the range seen in healthy controls (3.4-10.1 μg/mL). However, serum CFHR5 level was decreased in the index case (2.1 μg/mL). Notably, serum CFHR5 levels also were found to be decreased in individuals with biopsy-proven C3 glomerulonephritis (Fig 1D).

## Discussion

CFHR5 is a member of the CFH family of proteins encoded within the regulators of complement activation gene cluster on chromosome 1.^10^ CFHR5 has been shown to have complement-regulatory function in vitro^11^ and to colocalize with complement deposits within the kidney in a variety of glomerular pathologic states.^12^ The biological role of CFHR5 is unclear, but it is postulated to have a physiologic role in the processing of complement within the kidney.^13^ This hypothesis has derived from reports of genetic variation in CFHR5 and susceptibility to kidney disease. A heterozygous CFHR5 mutation has been shown to be associated with the development of familial C3 glomerulonephritis in individuals of Cypriot ancestry,^13^ and allelic variants of CFHR5 have been associated with dense deposit disease.^14^ Previously published evidence that CFHR5 interacts with glomerular complement^12,13^ suggests that this phenomenon contributed to the decrease in serum CFHR5 levels in the index case. Consistent with this hypothesis was our observation that glomerular CFHR5 was easily detectable in kidney biopsy specimens from the index case, median serum CFHR5 levels were low in individuals with C3 glomerulonephritis, and serum CFHR5 level in the index patient was lower than levels in other family members with the sequence variant, but no kidney disease. An interesting additional insight based on these data is that serum CFHR5 levels may be a useful biomarker of glomerular C3 deposition.

Our report is to our knowledge the first description of the association of kidney disease with a heterozygous sequence variant causing frameshift and premature truncation of CFHR5. Interestingly, the same variant that we describe was reported in an individual serving as a control in a study investigating the role of CFHR5 in atypical hemolytic uremic syndrome.^15^ No clinical details for this individual were reported. The presence of the CFHR5 sequence variant in the mother and sister of the index patient (neither of whom had overt evidence of kidney disease) indicates that heterozygosity for this CFHR5 sequence variant is not sufficient to cause kidney disease. We speculate that it was the presence of both the heterozygous CFHR5 sequence variant and streptococcal infection that precipitated chronic kidney disease in our index patient.

Interestingly, individuals with other genetic defects within the CFH gene family are susceptible to exacerbation of kidney disease during throat infections. Synpharyngitic macroscopic hematuria is characteristic of CFHR5 nephropathy^13^ and common in factor H deficiency states.^16,17^ Furthermore, exacerbation of urinary abnormalities in dense deposit disease has been associated with streptococcal infection.^18^ Rarely, kidney biopsies following APSGN have shown changes consistent with dense deposit disease.^19-21^ This raises the difficult question of whether dense deposit disease is triggered by streptococcal infection or is a pre-existing lesion that becomes clinically overt during streptococcal infection. We found one report describing apparent progression from biopsy-proven APSGN to dense deposit disease.^22^ However, in others, it seems that dense deposit disease may have predated the episode of APSGN.^18,20^ Our patient had no previous evidence of kidney disease, although it is possible that subclinical disease was present before the episode of streptococcal infection.

The presence of low serum C3 levels in this case cannot be explained by the heterozygous sequence variant in CFHR5 alone because serum C3 levels were normal in the index patient's mother and sister, who also carried the sequence variant. Notably, in CFHR5 nephropathy, serum C3 levels typically are normal.^13^ In contrast, in dense deposit disease, low serum C3 level is typical and commonly associated with the presence of C3NeF. However, rarer causes such as anti–factor B antibodies recently have been reported.^23^ C3NeF was repeatedly undetectable in our case and we could not detect any other factor within the serum of the index case that was causing accelerated C3 consumption. The serum C3 depletion in our case most likely results from excessive C3 activation within the kidney.

In summary, we have identified a heterozygous sequence variant causing frameshift and premature truncation of CFHR5 in an individual with chronic kidney disease after streptococcal infection. We speculate that CFHR5 mutations may be a risk factor for the development of chronic glomerulonephritis after streptococcal infection.

## Figures and Tables

**Table 1:** Complement Profile

	Pedigree No.					Reference Range
	I-2	II-1	II-2	II-4	II-3^a^	
CFHR5 sequence variant	Yes^b^	Yes^b^	No	No	Yes^b^	
C3 (g/L)	1.43	1.03	1.08	1.14	0.07	0.7-1.7
C4 (g/L)	0.24	0.17	0.21	0.16	0.22	0.16-0.54
CFH (%)^c^	181	132	132	147	148	
CFI (%)^c^	174	132	157	186	154	
Total complement hemolytic activity (%)^d^	108	47	99	95	ND	50-150
Alternative pathway hemolytic activity (%)^d^	92	53	102	95	ND	50-150
Serum CFHR5						
Absolute amount (μg/mL)	5.3	3.8	4.0	NA	2.1	3.4-10.1
Relative amount (%)^e^	96.2	69.9	72.5	NA	37.3	

Abbreviations: CFH, complement factor H; CFHR5, complement factor H-related protein 5; CFI, complement factor I; NA, not available (insufficient sample); ND, not detectable.

a Index patient.

b c.485dupA (p.Glu163Argfs*34); numbering and nomenclature follows Human Genome Variation Society recommendations (for cDNA, A in ATG start codon is position 1; for protein, the methionine encoded by the start codon is position 1).

c No validated reference range exists for CFH and CFI, so results are expressed as percentage of the level in pooled serum from apparently healthy controls.

d Percentage of the activity in pooled serum from apparently healthy controls.

e Compared with the median value in 13 apparently healthy controls, which was 5.5 μg/mL (Fig 1D).
